# How do study design features and participant characteristics influence willingness to participate in clinical trials? Results from a choice experiment

**DOI:** 10.1186/s12874-022-01803-6

**Published:** 2022-12-16

**Authors:** Caitlin Thomas, Sarah Mulnick, Nicolas Krucien, Kevin Marsh

**Affiliations:** 1 Evidera, The Ark, 201 Talgarth Road, London, W6 8BJ UK; 2 Evidera, Bethesda, MD USA

**Keywords:** Patient preference, Clinical trial design, Willingness to participate, Enrolment

## Abstract

**Background:**

Research about the decision to participate in a clinical study has tended to be limited to single indications and has focused on narrow sets of study and participant characteristics. This study applied stated preference methods to understand the clinical trial design attributes that most influence willingness to participate and how this varied with participant characteristics.

**Methods:**

Adults residing in the US, China, or Poland with a self-reported diagnosis of cancer, heart disease, migraine, rheumatoid arthritis, or multiple sclerosis completed an online survey. Participants were asked whether they would participate in clinical studies defined by seventeen attributes within five categories (payment/support, administration/procedures, treatment-related, study location/time commitment, and data collection/feedback). Participants saw six different hypothetical clinical study profiles. Depending on their participation decision to an initial clinical study profile, the subsequent five questions had one design attribute (randomly selected per question) consecutively improved or deteriorated to elicit preferences. A logistic regression was used to determine which participant characteristics influenced participation decisions. A latent class logit model was used to identify how the influence of study design features varied between participants and whether groups of participants with similar preferences could be identified.

**Results:**

The survey was completed by 487 participants (32% China, 35% Poland, 33% US; 8%–19% per indication). Willingness to participate was found to be a function of participant age, certain elements of quality of life, and previous treatment experience, in particular number of lines of treatment received and experience of adverse events. Willingness to participate was influenced by study design features such as payment, study duration, and time commitment – both the overall time and whether the time was at home or away from home, with the latter being particularly relevant to participants experiencing fatigue due to their disease.

**Conclusions:**

This study quantifies how study designs influence willingness to participate and how this varies with participant types. These findings suggest that it is how an indication influences quality of life and treatment experience, rather than the indication alone, that impacts participation rates, opening the way for insights that are transferrable across indications, which may be particularly useful when considering rare diseases.

**Supplementary Information:**

The online version contains supplementary material available at 10.1186/s12874-022-01803-6.

## Background

A successful clinical trial requires efficiently recruiting a sample of necessary size that is representative of the indication being assessed. However, over 80% of trials do not successfully enroll adequate samples within target timelines [1–3] and as many as 19% of clinical trials are terminated due to insufficient enrolment [4]. These shortcomings often necessitate adjustments such as extended timelines and additional sites to increase enrolment, which have important financial implications [2]. Studies also face challenges enrolling representative samples, resulting in greater proportions of younger, White, and male participants relative to target populations [5, 6]. Because treatment pharmacokinetics, efficacy, and safety can vary in racial and ethnic subpopulations [7, 8], representative and diverse participation is necessary to minimize outcome disparities between populations [9].

Few studies have investigated how willingness to participate varies with study design and participant characteristics; as such, little is known on how varying study design might improve enrolment rates and representativeness. Studies of willingness to participate in clinical research have largely been limited to single indications and have tended to focus on the influence of relatively narrow sets of participant characteristics, such as socio-demographic and attitudinal characteristics. The most commonly assessed characteristics include gender [10–14], age [10–16], and education level [11, 15, 17]. The lack of multi-indication studies also limits our understanding of how clinical characteristics influence participation. Research into the impact of disease on participation rates has been limited to proxies for treatment experience and stage of disease (e.g., initial treatment vs. retreatment and palliative vs. curative [13]). In addition, research has not considered how the impact of disease on quality and length of life, or how the type and availability of treatments for a disease influence willingness to participate. Trust in researchers, prior knowledge of clinical trials, and altruism have been associated with willingness to participate [14], although the impact of these factors on participation rates has, to the best of our knowledge, only been quantitatively assessed in a small number of cases [11, 17].

Some studies have quantified how trial design features impact willingness to participate, most commonly finding that participation increases with higher remuneration for participating [12, 18], lower risk of adverse events [10, 12], smaller time commitment associated with participating [18, 19], and involvement of a clinician in the trial or sharing of reports back with a clinician [18, 19]. However, this research does not help inform some of the key design questions currently being asked by study designers, such as how willingness to participate varies with strategies to decentralize trials (i.e., involving fewer hours at a clinical site and more hours at home), additional support for patients, and different methods of participant-completed data collection.

Enrolment into clinical trials could be improved through further understanding how willingness to participate varies with different clinical trial design attributes [20]. The objective of this study was to quantify the impact on willingness to participate of a comprehensive set of study characteristics and how this varies with participant characteristics.

## Methods

### Overview

An online stated-preference survey was conducted between March and April, 2021 with adult participants (≥ 18 years) with various indications (oncology specific, heart disease, migraine, rheumatoid arthritis, multiple sclerosis). The survey presented participants with a series of clinical study profiles that had desirable and undesirable study characteristics and asked them whether they would be willing to participate in these studies. The study profile information reflected key study information typically provided in an informed consent form. Participants with a wide range of indications were included to understand how the varied impact of a disease on current and future quality of life, as well as life expectancy influences willingness to participate. These indications also had varying levels of treatment availability, which we hypothesized may influence willingness to participate. At the end of the survey, participants were asked sociodemographic and clinical questions. The survey was programmed and hosted in EU Confirmit (Oslo, Norway) and was designed to take participants 30 min to complete; participants were told in advance the time required to complete the survey. The survey was conducted in the United States (US), Poland, and China to test how different healthcare systems, and potentially varied cultural views, influence willingness to participate. The online survey was translated from English into Mandarin and Polish by certified translators and validated by native-language Evidera employees. The study was approved by Ethical & Independent Review Services (E&I; Lee’s Summit, MO, US; E&I study number: 21038–01) and was conducted in accordance with best practice guidelines on preference-based methods from International Society for Pharmacoeconomics and Outcomes Research [21]. All participants provided online consent to participate. No personal information was collected. Results are reported in line with the recommendations outlined in the CHERRIES checklist for online surveys [22].

### Participants

Patients from the US (target: *n* = 160), Poland (target: *n* = 150), and China (target: *n* = 155) were identified and recruited by a third-party vendor (Global Perspectives, Norwich, UK) from patient panels. Patients were invited to participate in the study via emails that were generically worded to prevent biases in responsiveness. The emails contained a weblink to access the survey. To participate, patients had to self-report through a screening questionnaire on the first page of the online survey that they were aged ≥ 18 years; could read and understand the language that the survey was conducted in; and had received a diagnosis of lung cancer, colorectal cancer, liver cancer, stomach cancer, ovarian cancer, blood-related cancer, breast cancer, melanoma, prostate cancer, thyroid cancer, heart disease, migraine, rheumatoid arthritis, or multiple sclerosis. Patients also had to indicate that they were willing to consider participating in a clinical trial (responses: ‘yes’, ‘maybe’, ‘definitely not’); patients were excluded if they responded ‘definitely not’, since these patients would not be influenced by the design of a clinical trial. Eligible patients were then presented with the online consent form and after consenting progressed into the main survey. Target quotas were set for the different indications to ensure the sample was sufficiently diverse to allow differences in preferences between indications to be identified: *n* = 75 with higher mortality cancer (lung, colorectal, liver, stomach, ovarian, and blood cancers); *n* = 90 with lower mortality cancer (breast, melanoma, prostate, and thyroid cancers); *n* = 90 with heart disease; *n* = 85 with migraine; *n* = 85 with rheumatoid arthritis; *n* = 40 with multiple sclerosis. Higher- and lower-mortality cancers were defined based on mortality rates [23, 24].

### Selection of trial features

The features and levels that were used to define study profiles were identified based on a targeted literature review and two workshops with experts involved in clinical trial design. The targeted literature review was conducted within the search limits January 2000 to December 2020, using Embase and MEDLINE databases and with search strings related to adult patient preferences, motivation, and barriers to participating in clinical trials (Additional File 1). The search yielded 28 eligible articles, of which 20 were selected for extraction after excluding articles with significant overlap in concepts, prioritizing articles covering a wider range of indications, and prioritizing articles that quantitatively assessed impacts on willingness to participate. Several concepts were identified from the review which were grouped as (i) trial design and logistics; (ii) benefits and risks of participating; and (iii) attitude, knowledge, and social context influencing participation (Additional File 1: Figures S1–S3). The two workshops were undertaken in January and February, 2021 with 12 experts involved in clinical trial design at Pharmaceutical Product Development (PPD), Inc., who were recruited internally. The experts were specialized in various domains including oncology (*n* = 3), neurology (*n* = 3), diabetes, respiratory medicine, internal medicine, patient advocacy, and enterprise data, and digital and decentralized trial solutions (*n* = 1 for each). The aim of the workshops was to build on the literature to develop a comprehensive set of trial design features, levels, and patient characteristics that may influence willingness to participate. The final 17 trial design features and levels selected for inclusion in the study are shown in Table 1. Definitions of these features provided to participants are given in Additional File 2: Table S1. Some trial design features identified in the review and workshops were not included in the final list as they are invariably out of the control of the study design teams and were thus presented as fixed in preamble to the choice exercise. These included study funding by a pharmaceutical company, that participation may improve health, that studies are approved by an ethics committee or institutional review board, and that medication and care is provided free throughout the duration of the trial.

**Table 1:** Study design features and levels

Category	Clinical Study Feature	Levels
Payment and support	Payment you will receive ^a^	• $0 • $500 • $2,000*
	Transport	• Free transport is provided* • Prepaid card for travel • Transport costs can be claimed back
	Study Hours	• Study undertaken during normal office hours (9–5, Mon-Fri) • Option to participate out of office hours*
	Childcare	• Free childcare provided* • No childcare provided
	Concierge Service Provided	• Yes* • No
Administration / Procedures	Does treatment or study require an injection or infusion?	• Yes • No*
	Does treatment or study require an invasive procedure?	• No* • Minimally invasive procedure • Invasive procedure
Treatment-related	Number of participants who will have a serious side effect	• 0 out of 10 (0%)* • 2 out of 10 (20%) • 9 out of 10 (90%)
	Number of participants who will receive a placebo	• 0 out of 10 (0%, there is no placebo)* • 3 out of 10 (30%) • 5 out of 10 (50%)
	Is it possible to continue on treatment after trial?	• Yes* • No
	Are you required to stop using your current medications?	• Yes • No*
Study location and time commitment	Study duration	• 1 month* • 1 year • 2 years
	Time commitment per month at home	• 2 h / month* • 10 h / month • 30 h / month
	Time commitment per month away from home (i.e. time at study site and travel to site)	• 2 h / month* • 10 h / month • 30 h / month
Data collection and feedback	Does the study involve wearing a device?	• Yes • No*
	How will you provide self-reported data?	• Paper-based questionnaire • Electronic device*
	Will you be told the results of the study?	• No • Yes, the average results of everyone who took part • Yes, your own clinical results*

^*^These levels were the assumed ‘best’ level in the design. If a participant reported that they were not willing to participate in the trial shown in the first choice task, one attribute was improved to the assumed ‘best’ level and they were asked again whether they would participate

^a^Payment was shown to participants in local currency and calculated based on the same proportion for average salaries in China, Poland, and the US [25]. Poland payments: 0, 450, 1,800 zł. China payments: ¥0, 500, 2,000

### Stated preference exercise

**Fig. 1:**
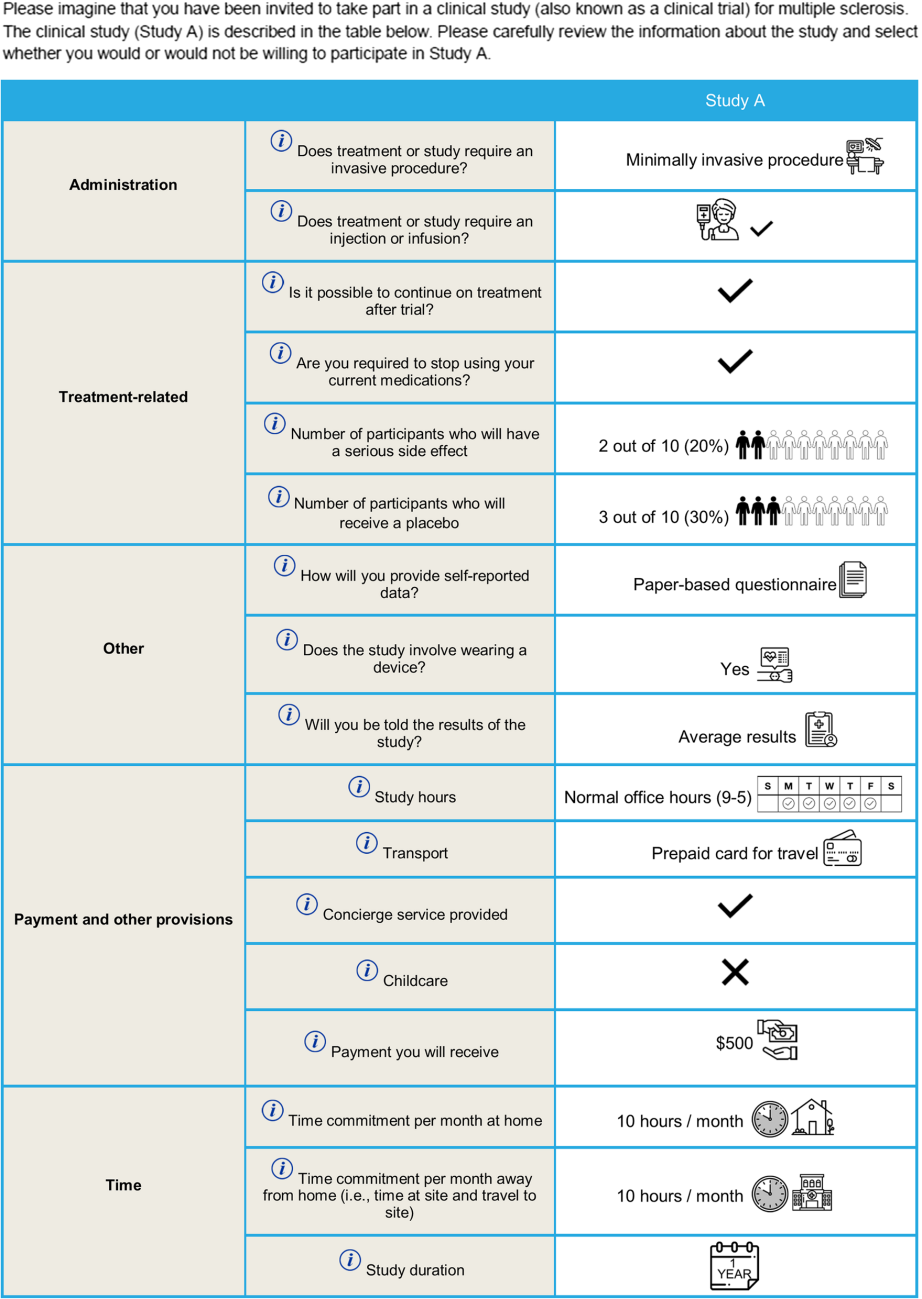
Example initial choice task (Study A) presented to participants

**Fig. 2:**
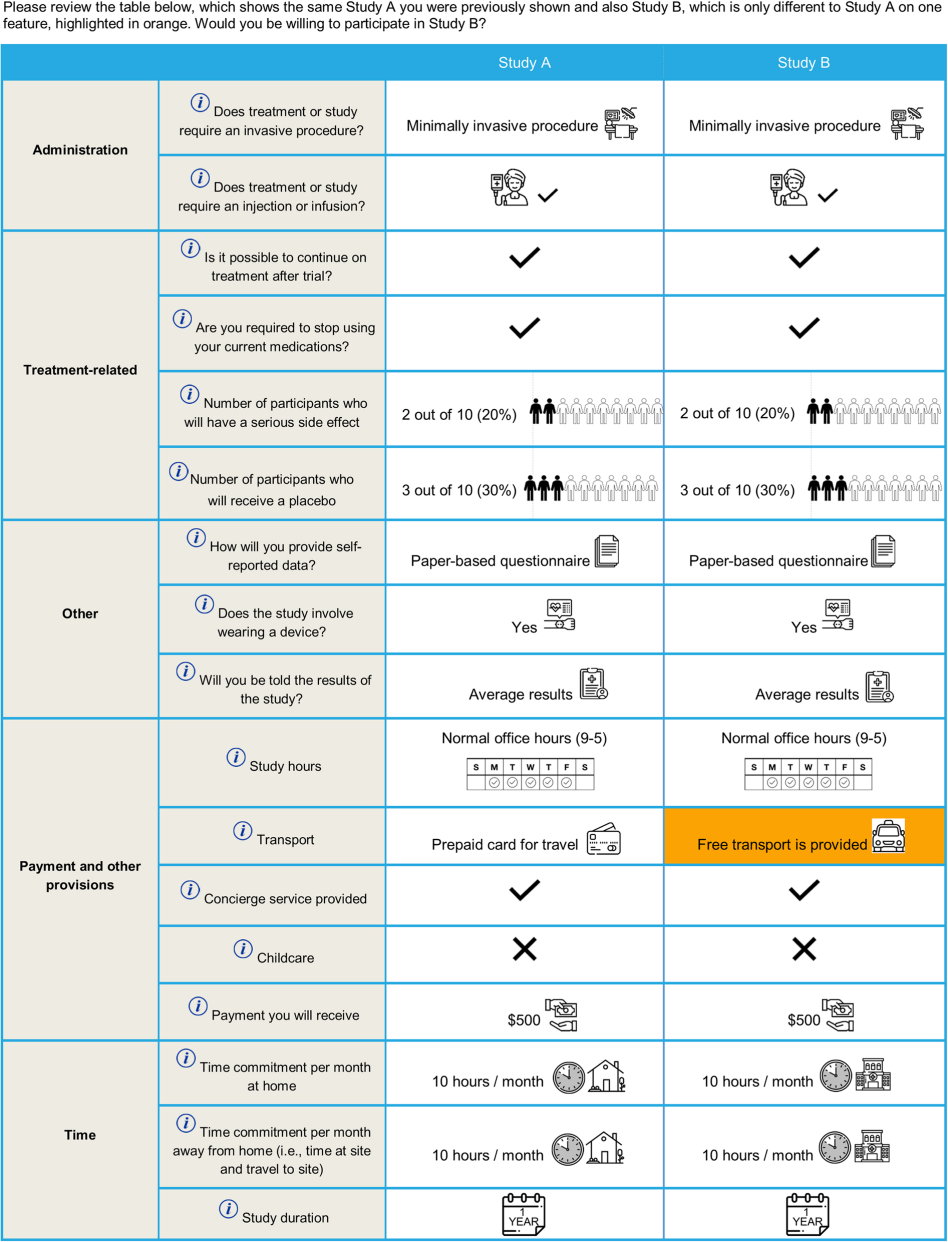
Example follow-up question showing one modified feature (highlighted)

After an introduction to the trial features (Additional File 2: Table S1) and the warm-up exercises, participants were presented with six choice tasks (one choice task per page); in each they were presented with a clinical trial design and asked whether they would be willing to participate in the hypothetical trial. The first choice task is shown in Fig. 1. Where a trial feature had three levels (e.g., “Payment you will receive”: $0, $500, $2,000), the first choice task used the middle level (i.e., $500). Where a feature had two levels, participants were randomized to see different levels of the feature in the first choice task. The survey was adaptive and answers to the initial choice question influenced whether trial features were improved or deteriorated in the five follow-up tasks. If a participant indicated that they were not willing to participate in the trial presented in the first choice task, the survey would improve the level of one randomly selected trial feature (one at a time) in the following five questions, and the participant was again asked whether they would participate (Fig. 2). Likewise, if a participant was willing to participate in the initial choice task, the survey would deteriorate one trial feature at a time in the following five questions. Modified features were highlighted for the participant. A different trial feature was deteriorated or improved in each case and this was randomized to measure preferences for all attributes. The order of the trial features were randomized throughout the study between participants to avoid ordering bias [26]. Participants were not able to review or change their answers. The survey was systematically validated prior to study start, including the screening logic and choice tasks. A researcher familiar with the survey tested the online programming using a practice link to ensure that the screening criteria was appropriately implemented, and ineligible participants were terminated from the screener. The choice task section was validated by reviewing each choice task to ensure that it was presented accurately according to the design.

### Measures of participant characteristics

At the end of the survey, participants were required to complete a series of sociodemographic and attitudinal questions, and questions about their illness. Questions adapted from the SF-36 [27], FACIT-FATIGUE [28] and EQ-5D [29] patient reported outcome measures were used to assess quality of life and the impact of illness on fatigue, social activity, mobility, ability to carry out daily activities, pain, and anxiety/depression. To assess trust and altruism, participants were asked to rate their agreement with items q3 and q4 of the Research Attitude Questionnaire [30] (q3: *Medical researchers can be trusted to protect the interests of people who take part in their studies*; q4: *We all have some responsibility to help others by volunteering for medical research*). Each were rated on a scale of 1 (strongly disagree) to 5 (strongly agree). Participants were considered to have high trust or high altruism if they rated their agreement as agree or strongly agree for each statement. Participants were also asked about prior clinical trial experience: whether they had been invited to participate in a trial, and whether they had participated in a trial or knew anyone who had participated in a trial.

### Statistical analysis

The choice task analysis was designed using Ngene software (ChoiceMetrics, Sydney, Australia), as described elsewhere [31–33], and other analyses were conducted using R (version 3.6.1). Only completed surveys were analyzed. Unless noted otherwise, all statistical tests were two-sided and used a significance level of 0.05. No adjustments for multiple comparisons were performed unless noted otherwise. A logistic regression was used to identify patient characteristics that influenced the decision to participate in the initial trial presented. Three variations of the logistic regression were conducted, each including different patient characteristic variables, to assess how accurately decisions could be predicted if different variations of patient characteristics were known: Model 1 – all patient characteristic variables included (i.e., disease-related variables, treatment-related variables, sociodemographic variables, and attitudinal variables); Model 2 – only disease-related variables and country of residence included; Model 3 – all variables excluding disease-related variables and country of residence. The three variants were manually defined to represent cases where the trial designer would have incomplete information about individuals’ characteristics. For the logistic regression analysis, we combined participants with 1–2 conditions. Pearson correlations among the selected covariates were computed to avoid potential collinearity issues (covariates with an absolute correlation larger than 0.8 were excluded from the analysis [34]). Several criteria were used to evaluate the statistical performance of the different models: Bayesian Information Criteria (BIC) and out-of-sample predictive validity (i.e., % of unseen choices correctly predicted by the model). The out-of-sample predictive validity was obtained with a tenfold cross-validation procedure.

A latent class logistic (LCL) model [35] was estimated on the five follow-up tasks to analyze the likelihood that participants would change their initial decision to participate as features were varied. The optimal number of latent classes (probabilistic groups with distinct preferences), was determined by estimating LCL models with 2 to 5 classes. Each model was estimated with 20 different sets of starting values to increase the chance of finding the best solution (i.e., global maxima). The final number of classes was selected by balancing goodness-of-fit with the number of included parameters using the BIC and interpretability (i.e., the classes have logical and clinical relevance). Odds ratios (ORs) were used to report the effect of changes in the clinical trial design features on the decision to participate relative to changes in one feature – transport provision. The LCL estimates were then used to compute the class membership probabilities (i.e., probability of each participant falling into the different classes defined in the LCL model). The variability in these probabilities was then analyzed in a Beta regression model as a function of respondents’ personal characteristics.

## Results

### Participant characteristics

From approximately 150,000 invitation emails, 8,950 individuals visited the first page of the survey (view rate, 0.06%; Additional File 3: Figure S4). Of these, 8,275 individuals (92.5%) were not eligible (i.e., did not consent, were screened out, or quota full); this included 58 of 545 otherwise eligible participants (10.6%) who responded that they would ‘definitely not’ consider participating in a clinical trial. Ultimately, 561 were eligible and provided online consent (participation rate, 0.06%). The survey was completed by 487 participants (completion rate, 86.8%). The mean age was 47 ± 17 years (Table 2). Three-quarters (*n* = 360, 74%) of participants had a college education or higher, more than half (*n* = 276, 57%) were employed 30 h or longer per week, and a third (*n* = 161, 33%) were not working in a paid job. Most participants (*n* = 412, 85%) said that they trusted medical researchers and most (*n* = 404, 83%) were altruistic – as indicated by interest in volunteering for medical research. Migraine and rheumatoid arthritis were the most represented indications, followed by heart disease (Table 3). Two hundred participants reported one of the pre-specified medical conditions, 115 reported two, and 172 reported three or more. Most participants (*n* = 300, 61%) had taken ≥ 3 treatments for their condition and most could afford or easily afford (*n* = 440, 90%) their current medications. Most participants rated their health as good or fair (*n* = 366, 75%). There were large correlations (Cohen’s coefficient, 0.5–0.7) identified between country and age group (0.63), employment status and age group (0.61), and employment status and country (0.54) (Additional File 4: Table S2). Additional participant demographics and baseline characteristics are given in Additional File 5: Table S3. The median response time [10^th^–90^th^ percentile] for the choice task part of the survey did not significantly differ across the three countries (Brown-Mood Median Test: chi-square = 5.3, *p* = 0.071): USA = 12.7 min [7.6–26.5]; Poland: 12.2 min [6.7–26.5]; China: 11.3 min [5.5–39.8].

**Table 2:** Sociodemographic characteristics and attitudes towards medical research (*N* = 487)

Characteristic	Value
Country of residence, n (%)	
United States	161 (33)
Poland	169 (35)
China	157 (32)
Sex, n (%)	
Female	253 (52)
Age (years)	
Mean (SD)	47 (17)
Min–Max	19 – 91
Age group, n (%)	
18–35 years	152 (31)
36–50 years	138 (28)
51–65 years	82 (17)
≥ 66 years	115 (24)
Educational level ^a^, n (%)	
Less than elementary/primary school	1 (0)
Elementary/primary school	75 (15)
High school/technical school or equivalent	49 (10)
College / University degree (BA, BSc)	242 (50)
Post-graduate degree (MA, PhD)	118 (24)
Other	2 (0)
Employment status, n (%)	
Employed ≥ 30 h per week with paid time off	276 (57)
Employed < 30 h per week with paid time off	31 (6)
Employed on hourly basis (i.e., not a fixed salary) with no paid time off	19 (4)
Not working in a paid job (i.e. homemaker, voluntary work, retired, student, unemployed, not able to work due to sickness)	161 (33)
Experience of taking part in a clinical trial, n (%)	
Experience (self or relative)	160 (33)
No experience	327 (67)
Trust in medical researchers ^b^, n (%)	
High trust (agree/strongly agree)	412 (85)
Low trust (disagree / strongly disagree)	75 (15)
Altruism ^c^, n (%)	
High altruism (agree/strongly agree)	404 (83)
Low altruism (disagree / strongly disagree)	83 (17)

^a^ Less than elementary/primary school education includes *no formal qualifications*; Elementary / primary school = less than high school/technical school or equivalent; High school / technical school or equivalent includes post-secondary

^b^ Trust in medical researchers assessed by asking participants to rate their agreement with item q3 of the Research Attitude Questionnaire [30]: ‘*Medical researchers can be trusted to protect the interests of people who take part in their studies*’

^c^ Altruism assessed by asking participants to rate their agreement with item q4 of the Research Attitude Questionnaire [30]: ‘*We all have some responsibility to help others by volunteering for medical research*’

**Table 3:** Clinical characteristics (*N* = 487)

Characteristic	Value
Diagnosed indication ^a^, n (%)	
Cancer	183 (38)
*Higher mortality cancers*	*87*
Lung cancer	22 (12)
Colorectal cancer	12 (7)
Liver cancer	12 (7)
Stomach cancer	3 (2)
Ovarian cancer	22 (12)
Multiple myeloma	6 (3)
Leukemia or another blood-related cancer	15 (8)
*Lower mortality cancers*	*96*
Breast cancer	39 (21)
Melanoma / skin cancer	23 (13)
Prostate cancer	32 (17)
Thyroid cancer	9 (5)
Heart disease	122 (25)
Migraine	178 (37)
Multiple sclerosis	51 (10)
Rheumatoid arthritis	134 (28)
Anemia	93 (19)
Anxiety	111 (23)
Depression	106 (22)
Diabetes (Type I or Type II)	106 (22)
Number of medical conditions ^b^, n (%)	
1	200 (41)
2	115 (24)
≥ 3	172 (35)
Number of previous lines of treatment, n (%)	
0 treatment	41 (8)
1–2 treatments	146 (30)
3–4 treatments	132 (27)
≥ 5 treatments	168 (34)
Treatment options are available for condition, n (%)	
There are not many alternative treatments available	98 (20)
There are some alternative treatments available	203 (42)
There are lots of alternative treatments available	115 (24)
Don't know	71 (15)
Affordability of current medication	
I can easily afford my medications	168 (34)
I can afford my medications	272 (56)
I struggle to pay for my medications	47 (10)
Experience of treatment side effects, n (%)	
I have not experienced any side effects	155 (32)
I have experienced mild side effects	249 (51)
I have experienced severe side effects	83 (17)
Satisfaction with current disease management, n (%)	
Not at all satisfied	69 (14)
Moderately satisfied	263 (54)
Very much satisfied	155 (32)
Quality of Life (Classification) ^c^	
0–3	106 (22)
4–7	128 (26)
8–11	103 (21)
12–15	87 (18)
16–19	44 (9)
20–24	19 (14)
I feel fatigued	
Not at all / A little bit	209 (43)
Somewhat	129 (26)
Quite a bit / Very much	149 (31)
I have had difficulty with mobility	
Not at all / A little bit	336 (69)
Somewhat	84 (17)
Quite a bit / Very much	67 (14)
I have had pain or discomfort	
Not at all / A little bit	244 (50)
Somewhat	121 (25)
Quite a bit / Very much	122 (25)
I have been anxious or depressed	
Not at all / A little bit	276 (57)
Somewhat	87 (18)
Quite a bit / Very much	124 (25)
I have to limit my social activity because of my health	
Not at all / A little bit	282 (58)
Somewhat	98 (20)
Quite a bit / Very much	107 (22)
I have had difficulty with performing my usual activities	
Not at all / A little bit	310 (64)
Somewhat	107 (22)
Quite a bit / Very much	70 (14)
Self-perceived health status, n (%)	
Excellent or Very Good	71 (15)
Good	176 (36)
Fair	190 (39)
Poor	50 (10)
Self-perceived likelihood to live to average age ^d^, n (%)	
Likely / Highly likely	261 (54)
Neutral	125 (26)
Highly unlikely / Unlikely	80 (16)
Not sure / Don't know	21 (4)
Self-perceived future quality of life, n (%)	
Much better / Somewhat better in 10 years than now	91 (19)
About the same in 10 years than now	145 (30)
Somewhat worse / Much worse in 10 years than now	251 (52)

^a^ Not mutually exclusive. Denominator used for percentages is the overall sample *n* = 487, except for cancer subtypes where the denominator is the number reporting any cancer diagnosis (*n* = 183)

^b^ Of the specified diagnosed indications only

^c^ The 6 quality-of-life domain-related questions (fatigue, social activity, mobility, impact on usual activities, discomfort, anxiety/depression) were rated on 0–4-point rating scales (where 0 = not at all, and 4 = very much). Total scores were computed (ranging from 0–24) and then classified into one of three groups: 0–7; 8–16; and 17–24

^d^ Participants asked the likelihood they will live for the next 50 years if aged 18–35, the next 40 years if aged 36–50, the next 25 years if aged 51–65, or the next 10 years if aged ≥ 66

### Participant characteristics influencing willingness to participate

In the initial choice task, most participants (*n* = 344, 71%) indicated that they were willing to participate in the clinical study presented to them (i.e., ‘Study A’) (Fig. 1). Table 4 reports the logistic regression results of the initial participation decision. When all participant characteristics were considered (Model 1), participants were less likely to participate in Study A if they were diagnosed over 6 years ago or had depression, and were more likely to participate if they had previously experienced treatment side effects, had a lower self-perceived life expectancy, experienced disease impacts on their social activities, or were more altruistic (*p* < 0.1 for all). Participants’ country of residence also affected willingness to participate, with those in China more likely to participate than those in the US (*p* < 0.01), although there was no significant difference in willingness to participate between participants in Poland and those in the US.

**Table 4:** Participant characteristics predicting willingness to participate (logistic regression results)

Characteristics	Reference	Levels	MLE (SE) ^a^		
			**Model 1: All**	**Model 2: Disease & Country**	**Model 3: All except disease & country**
**Constant**		-	-1.042 (0.873)	-0.067 (0.334)	-0.286 (0.652)
**Disease-related variables**					
Time since first diagnosis, years	0–5	≥ 6	**-0.498 (0.277)** ^*^	**-0.527 (0.240)** ^**^	-
Number of medical conditions	1–2	≥ 3	0.850 (0.547)	**0.794 (0.481)** ^*^	-
Anemia	No	Yes	0.100 (0.417)	0.038 (0.365)	-
Anxiety	No	Yes	-0.007 (0.399)	-0.002 (0.357)	-
Depression	No	Yes	**-0.808 (0.445)** ^*^	-0.491 (0.380)	-
Diabetes (Type I or Type II)	No	Yes	-0.206 (0.354)	-0.024 (0.304)	-
Heart disease	No	Yes	-0.394 (0.352)	-0.040 (0.312)	-
Migraine	No	Yes	0.300 (0.358)	0.363 (0.321)	-
Multiple sclerosis	No	Yes	0.130 (0.492)	0.404 (0.431)	-
Rheumatoid arthritis	No	Yes	-0.022 (0.332)	0.179 (0.303)	-
Cancer	No	Lower mortality ^b^	0.055 (0.413)	0.300 (0.345)	-
	No	Higher mortality ^c^	-0.103 (0.407)	0.249 (0.357)	-
**Treatment-related variables**					
Number of previous lines of treatment	0–2	≥ 3	0.320 (0.276)	-	**0.531 (0.251)** ^**^
Affordability of current medication	I can easily afford my medications	I can afford my medications	-0.357 (0.288)	-	-0.276 (0.263)
	I can easily afford my medications	I struggle to pay for my medications	-0.224 (0.501)	-	-0.020 (0.467)
Experience of treatment side effects	None	Mild	**0.558 (0.288)** ^*^	-	**0.594 (0.271)** ^**^
	None	Severe	0.756 (0.431)^*^	-	0.753 (0.390)^*^
COVID vaccine	No	Yes	0.269 (0.332)	-	-
Direct experience of clinical trial	No	Yes	0.409 (0.403)	-	-
**Health-related variables**					
I feel fatigued	Not at all/ A little bit	Somewhat/Quite a bit/Very Much	0.192 (0.335)	-	-0.025 (0.288)
I have had difficulty with mobility	Not at all/ A little bit	Somewhat/Quite a Bit/Very Much	0.115 (0.375)	-	0.168 (0.329)
I have had pain or discomfort	Not at all/ A little bit	Somewhat/Quite a Bit/Very Much	-0.120 (0.337)	-	0.115 (0.288)
I have been anxious or depressed	Not at all/ A little bit	Somewhat/Quite a Bit/Very Much	-0.551 (0.375)	-	-0.553 (0.303)^*^
I have to limit my social activity because of my health	Not at all/ A little Bit /Somewhat	Quite a Bit/Very Much	0.795 (0.410)^*^	-	0.808 (0.384)^**^
I have had difficulty with performing my usual activities	Not at all/A little Bit /Somewhat	Quite a Bit/Very Much	-0.577 (0.462)	-	-0.704 (0.435)
Current QoL (Classification) ^d^	0–7	≥ 8	0.151 (0.465)	-	-
QoL vs. last year	Improve	Same/Deteriorate	-0.213 (0.315)	-	-0.393 (0.289)
Self-perceived likelihood to live to average age ^e^	Neutral/High	Low	0.716 (0.367)^*^	-	0.266 (0.345)
Self-perceived future quality of life	Improve	Same/Deteriorate	0.048 (0.427)	-	0.035 (0.389)
**Sociodemographic variables**					
Age, years	18–50	51–65	-0.459 (0.401)	-	-1.014 (0.355)^***^
	18–50	≥ 66	-0.916 (0.557)	-	-1.779 (0.427)^***^
Sex at birth	Male	Female	-0.177 (0.263)	-	-0.158 (0.243)
Country of residence	USA	Poland	0.482 (0.471)	0.759 (0.249)^***^	-
	USA	China	1.899 (0.516)^***^	2.162 (0.357)^***^	-
Paid job	Yes	No	-0.105 (0.354)	-	-0.198 (0.334)
Education	Other than university	University	-0.468 (0.318)	-	-0.162 (0.286)
Living alone	Yes	No	0.116 (0.373)	-	0.138 (0.347)
Looking after dependent(s)	Yes	No	0.250 (0.318)	-	0.198 (0.294)
**Attitudinal variables**					
Trust in medical researchers ^f^	Low (1–3)	High (4–5)	0.275 (0.341)	-	0.523 (0.327)
Altruism ^g^	Low (1–3)	High (4–5)	1.074 (0.341)^***^	-	1.017 (0.321)^***^
**Other variables**					
Survey version	Version 1	Version 2	0.979 (0.261)^***^	-	0.825 (0.242)^***^
**BIC**			695.2	590	625.3
**PV**			72.92%	71.88%	73.54%

Abbreviations: BIC, Bayesian Information Criterion; CP, % of correctly predicted choices; MLE, maximum likelihood estimation; PV, % of unseen choices correctly predicted (holdout sample); QoL, quality of life

^*^p < 0.1; **p < 0.05; ***p < 0.01

^a^ The constant is the reference group of all dummy variables (all dummy variables = 0). Each dummy coded variable coefficient represents the difference from the reference

^b^ Lower mortality cancer = breast cancer, melanoma / skin cancer, prostate cancer, thyroid cancer

^c^ Higher mortality cancer = colorectal cancer, leukemia or another blood-related cancer, liver cancer, lung cancer, multiple myeloma, ovarian cancer, stomach cancer

^d^ The 6 QoL domain-related questions (fatigue, social activity, mobility, impact on usual activities, discomfort, anxiety/depression) were rated on 0–4-point rating scales (where 0 = Not at all, and 4 = Very much). Total scores were computed (ranging from 0–24) and then classified into one of three groups: 0–7; 8–16; and 17–24

^e^ Participants asked the likelihood they will live for the next 50 years if aged 18–35, the next 40 years if aged 36–50, the next 25 years if aged 51–65, or the next 10 years if aged ≥ 66

^f^ Trust in medical researchers assessed by asking participants to rate their agreement with item q3 of the Research Attitude Questionnaire [30]: ‘*Medical researchers can be trusted to protect the interests of people who take part in their studies*’. Rated on a scale of 1 (strongly disagree) to 5 (strongly agree)

^g^ Altruism assessed by asking participants to rate their agreement with item q4 of the Research Attitude Questionnaire [30]: ‘*We all have some responsibility to help others by volunteering for medical research*’. Rated on a scale of 1 (strongly disagree) to 5 (strongly agree)

The modelling performance, measured with BIC and predictive accuracy, remained similar when the model was run with only disease-related characteristics and country (Model 2) or with all characteristics excluding disease-related characteristics and country (Model 3) (Table 4).

In Model 2, the participant characteristics that influenced the initial decision to participate were time since diagnosis (*p* < 0.05) and country (*p* < 0.01), similar to Model 1, but also the number of medical conditions (*p* < 0.1); those with three or more medical conditions were more likely to be willing to participate.

In Model 3, the participant characteristics that influenced the initial decision to participate were the number of previous lines of treatment (*p* < 0.05), experience of treatment side effects (*p* < 0.05), feeling anxious or depressed (*p* < 0.1), experience of disease impacts on social activities (*p* < 0.05), age (*p* < 0.01), and altruism (*p* < 0.01). With the exception of experience of side effects and altruism, these participant characteristics emerged either as being relevant to understanding willingness to participate or their effect was found to be significant once indication was no longer controlled for in the model.

### Trial design features influencing willingness to participate

When the levels of trial design features were altered (i.e., one feature changed at a time in the five follow-up tasks), participants changed their initial decision to participate in 25% of questions. Less than half (42%) of participants always maintained their initial participation decision and 3% changed their decision in all five follow-up questions.

**Fig. 3:**
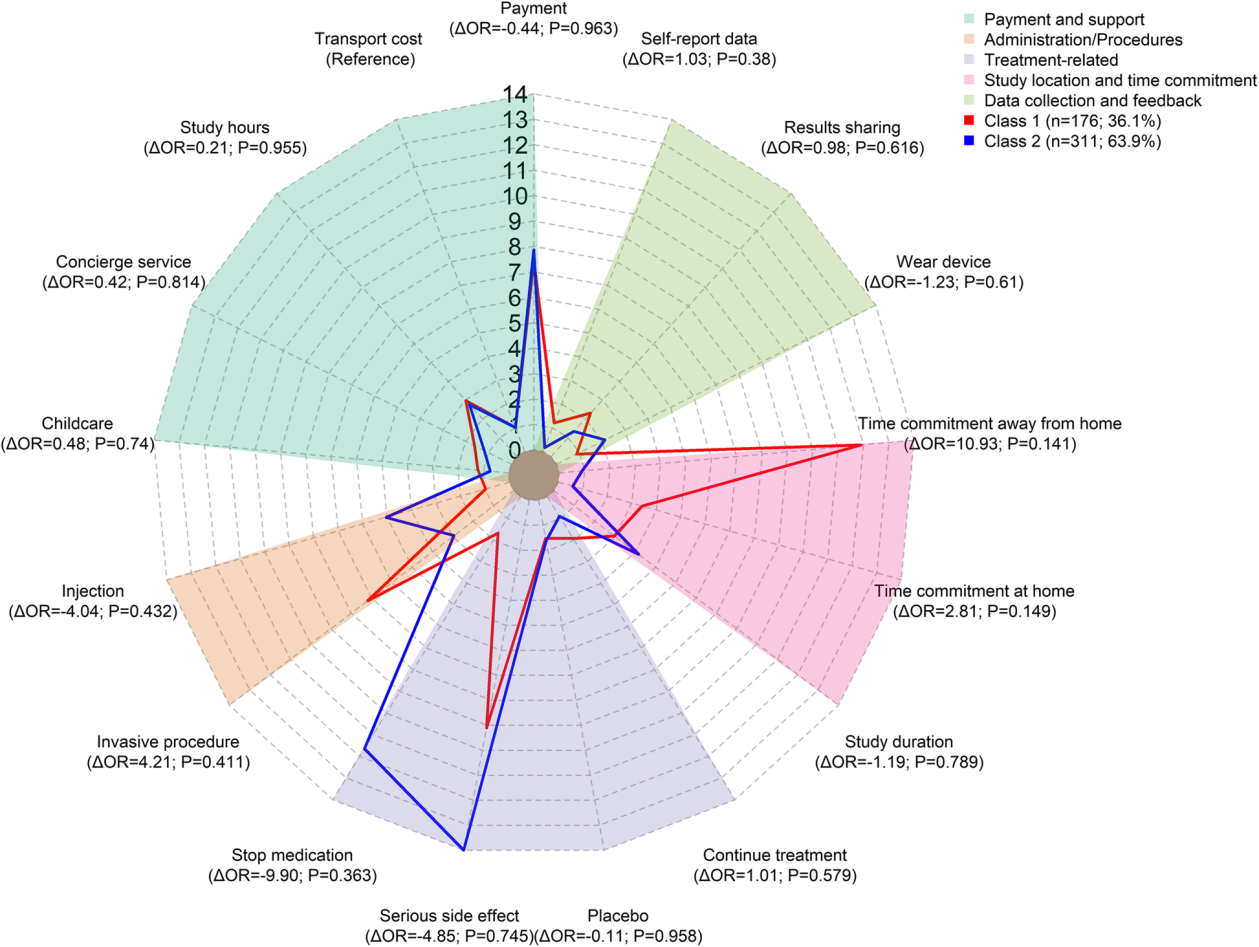
Class Preferences for Trial Design Features (Latent Class Results). The odds ratios (OR) report the relative likelihood that participants in each class would change their decision to participate as a feature is varied relative to the impact on decisions of changes in transport provision

When assessing which trial design features influenced willingness to participate, the latent class model identified two groups (classes) of participants, which provided the best balance between model fit and interpretability (Fig. 3 and Additional File 6: Table S4). Class 1 (36.1%) were influenced by several trial design features, whereas Class 2 (63.9%) were more concerned with treatment characteristics and less influenced by changes in trial design other than payment for participating. When trial design features were altered, Class 2 changed their initial decision to participate on fewer occasions than Class 1 (8% vs. 55%).

Relative to changes in transport provision, Class 1 were more likely to change their participation decision than Class 2 when the following design features were varied: time commitment away from home (OR, 11.83 vs. 0.90), having an invasive procedure (OR, 7.13 vs. 2.92), time commitment at home (OR, 3.40 vs. 0.58), and level of results-sharing with participants (OR, 2.29 vs. 1.32). These attributes did not have a significant impact on the participation decisions of Class 2. For Class 1, time commitment away from home was of greater concern than time commitment at home (OR, 11.83 vs. 3.40).

Relative to changes in transport provision, Class 2 cared more than Class 1 about the requirement to stop existing medication for their condition (OR, 11.56 vs. 1.66).

Two characteristics had a high impact on participation decisions of both classes relative to the impact of changing transport provision: payment (Class 1 OR, 7.37; Class 2 OR, 7.81) and risk of serious side effects (Class 1 OR, 9.04; Class 2 OR, 13.89).

For both classes, relative to transport provision, participation decisions were relatively less influenced by variation in childcare provision, having a concierge service, the ability to continue treatment after trial, likelihood of placebo, the requirement to wear a device, or the requirement to self-report data electronically vs. on paper.

Class allocation was influenced by participant characteristics (Table 5). Those who were older (≥ 66 years old relative to 18–50 years old) and those whose condition impacted their ability to engage in social activities were more likely to be in Class 2 – the class that was more concerned by having to stop their existing medication, and were also more concerned by avoiding side effects. Those who had received more lines of treatment, those who struggle to afford their medications, and those who experienced fatigue from their condition were more likely to be in Class 1 – the class that was more concerned by how the study design would impact the location of study activities (e.g., at or away from home).

**Table 5:** Class allocation – likelihood of being in Class 2 over Class 1

Characteristics	Reference level	Levels	MLE (SE)
**Constant**	-	-	-0.283 (0.432)
**Disease-related variables**			
Time since first diagnosis, years	0–5	≥ 6	-0.156 (0.140)
Number of medical conditions	1–2	≥ 3	-0.042 (0.247)
Anemia	No	Yes	-0.032 (0.184)
Anxiety	No	Yes	0.138 (0.191)
Depression	No	Yes	-0.169 (0.200)
Diabetes (Type I or Type II)	No	Yes	0.222 (0.167)
Heart disease	No	Yes	0.037 (0.166)
Migraine	No	Yes	0.215 (0.171)
Multiple sclerosis	No	Yes	0.191 (0.222)
Rheumatoid arthritis	No	Yes	0.013 (0.159)
Cancer	No	Lower mortality	-0.087 (0.206)
	No	Higher mortality	0.126 (0.199)
**Treatment-related variables**			
Number of previous lines of treatment	0–2	≥ 3	-0.304 (0.146)^**^
Affordability of current medication	I can easily afford my medications	I can afford my medications	-0.200 (0.142)
	I can easily afford my medications	I struggle to pay for my medications	-0.403 (0.245)^*^
Experience of treatment side effects	None	Mild	0.097 (0.149)
	None	Severe	0.024 (0.210)
COVID vaccine	No	Yes	-0.062 (0.152)
Direct experience of clinical trial	No	Yes	0.245 (0.188)
**Health-related variables**			
I feel fatigued	Not at all/A little Bit	Somewhat/Quite a Bit/Very Much	-0.288 (0.165)^*^
I have had difficulty with mobility	Not at all/A little Bit	Somewhat/Quite a Bit/Very Much	-0.021 (0.178)
I have had pain or discomfort	Not at all/A little Bit	Somewhat/Quite a Bit/Very Much	0.053 (0.165)
I have been anxious or depressed	Not at all/A little Bit	Somewhat/Quite a Bit/Very Much	0.126 (0.170)
I have to limit my social activity because of my health	Not at all/A little Bit/Somewhat	Quite a Bit/Very Much	0.372 (0.184)^**^
I have had difficulty with performing my usual activities	Not at all/A little Bit/Somewhat	Quite a Bit/Very Much	0.042 (0.215)
Current QoL (Classification)	0–7	≥ 8	0.276 (0.222)
QoL vs. last year	Improve	Same/Deteriorate	0.098 (0.149)
Self-perceived likelihood to live to average age ^a^	Neutral/High	Low	0.096 (0.184)
Self-perceived future quality of life	Improve	Same/Deteriorate	-0.130 (0.185)
**Sociodemographic variables**			
Age, years	18–50	51–65	0.279 (0.209)
	18–50	≥ 66	0.946 (0.299)^***^
Sex at birth	Male	Female	0.068 (0.130)
Country of residence	USA	Poland	0.218 (0.245)
		China	0.195 (0.242)
Paid job	Yes	No	-0.213 (0.190)
Education	Other than university	University	-0.168 (0.163)
Living alone	Yes	No	0.279 (0.191)
Looking after dependent	Yes	No	0.094 (0.154)
**Attitudinal variables**			
Trust in medical researchers ^b^	Low (1–3)	High (4–5)	-0.035 (0.186)
Altruism ^c^	Low (1–3)	High (4–5)	-0.020 (0.179)
**Other variables**			
Survey version	Version 1	Version 2	0.154 (0.124)

*Abbreviations*: *MLE* maximum likelihood estimation, *QoL* quality of life, *SE* standard error

^*^p < 0.1; **p < 0.05; ***p < 0.01

^a^ Participants asked the likelihood they will live for the next 50 years if aged 18–35, the next 40 years if aged 36–50, the next 25 years if aged 51–65, or the next 10 years if aged ≥ 66

^b^ Trust in medical researchers assessed by asking participants to rate their agreement with item q3 of the Research Attitude Questionnaire [30]: ‘*Medical researchers can be trusted to protect the interests of people who take part in their studies*’. Rated on a scale of 1 (strongly disagree) to 5 (strongly agree)

^c^ Altruism assessed by asking participants to rate their agreement with item q4 of the Research Attitude Questionnaire [30]: ‘*We all have some responsibility to help others by volunteering for medical research*’. Rated on a scale of 1 (strongly disagree) to 5 (strongly agree)

## Discussion

The objective of this study was to quantify the impact of differing study characteristics on willingness to participate and how this varies with participant characteristics. The results of this study suggest that participation rates are a function of how an indication influences quality of life as well as a patient's treatment experience, not the indication itself. Further, participation rates can be influenced by study design features such as payment, study duration, and amount and location of time commitment, with the latter being particularly relevant to participants who are experiencing fatigue due to their disease.

In line with earlier studies, participants who were more altruistic or who had received more treatments were more willing to participate in clinical studies [13, 14, 17] and participants who were older were less willing to participate [10–12, 15]. It is unclear from prior quantitative studies whether willingness to participate is influenced by gender [10, 11, 14], education [10, 15], affordability of medications [15], or trust in medical researchers [12] due to variability in results and a lack of evidence for these characteristics. In our study, these characteristics were less predictive of willingness to participate. Several participant characteristics not considered in previous studies were found to influence willingness to participate, including the impact of disease on elements of quality of life, especially their social life and whether they were anxious or depressed; and whether patients had experienced adverse events with their existing treatments. Several other characteristics that we hypothesized might predict willingness to participate were not found to influence participation, such life expectancy (self-perceived), indication, and employment status.

Willingness to participate differed between countries. Specifically, Chinese participants had higher willingness to participate than US participants. Such country effects have also been found in other studies; for example, those with a Swiss nationality were found to have higher willingness to participate than those from European and other countries [10]. However, it may be that such effects reflect differences in the samples between countries, rather than being a genuine country effect. In our study, country was correlated with other participant characteristics such as age and employment status. Further, the predictive validity of the models were not reduced when country was removed from the model, with other participant characteristics then proving more relevant, such as the number of prior lines of therapy, experience of treatment side effects, impact of disease on quality of life (social activities and anxiety/depression), age, and altruism. This suggests that it is variation between countries in these characteristics, rather than the country itself, that drive participation decisions.

All participants were influenced by a number of study design features, including: study duration and, mirroring other studies [10, 12, 18, 36], the amount of payment and the likelihood of experiencing adverse events. The influence of other study design features varied between participants. Participants who were older and whose disease impacted their social activities were more concerned with having to stop existing medication to participate, as well as being more concerned about side effects. Other participants, particularly those whose disease caused them to feel fatigued, were concerned with the time commitment associated with being in a study, especially if that was away from home. This cohort may be more likely to participate in de-centralized studies, implementation of which has accelerated in recent years – largely due to the COVID-19 pandemic [37]. Clinical trial decentralization is expected to be a long-term trend that may ease the burden of participating and potentially improve recruitment and retention of diverse participants [38]. Our results help to understand which populations this may be most effective in. In contrast to other studies [10, 18], willingness to participate was not significantly influenced by the likelihood of participants being in the placebo group or whether the trial involved an open label extension, which may be due to our study controlling for a greater number of study characteristics.

Our study had limitations. First, the study included many trial design features, which could have made the choice tasks cognitively burdensome. To mitigate this, the initial choice task adopted a single profile format asking whether participants would participate in this one trial (yes/no), and follow-up choice tasks varied only one feature at a time (highlighted to the participant). While this design helped to limit complexity, it also limited the amount of information gathered on trade-offs and the ability to test interactions between design characteristics. Second, it was not possible to conduct cognitive interviews to test that the concepts were being interpreted similarly across the three countries studied. Further research could usefully include this step. Third, recruitment quotas focused only on indication. Whilst the sample was diverse by country, gender, and age, it was not diverse in ethnic or racial background within each country; therefore, it was not possible to investigate whether ethnicity or race influenced willingness to participate. Fourth, participants self-reporting their diagnosis rather than it being clinically confirmed, and recall bias could have affected participants’ responses to the clinical and sociodemographic questions. Fifth, participants were sampled from an opt-in panel of individuals who signed up to participate in health care research studies; therefore, willingness to participate may be overestimated. Professional and established access panels were used to mitigate risks that might have resulted from recruitment via self-reported diagnosis. Sixth, hypothetical bias is inherent in stated preference research, since participants are asked to make hypothetical choices that may not reflect their actual decisions if they were invited to a clinical trial [39]. Although external validity of stated preference data has been demonstrated under certain conditions [40], further work is required to test the external validity of stated preference research. Finally, at the screening stage, patients who would ‘definitely not’ participate in any form of clinical trial were excluded – corresponding to 10% of the otherwise eligible participants. This aligns with similar research showing 10.3% would ‘certainly refuse’ to take part in a fictive trial of a new respiratory drug [36], although we expect the rate to vary between indications and countries. While our study did not explore the reasons behind this choice (e.g., with follow-on questions), understanding why some individuals will never participate in clinical trials is an important avenue for future research. This may correspond to attitudinal factors among patients, such as altruism and trust [14, 16, 17].

Our work has generated preliminary insights into which trial design features influence patients’ decisions to participate in clinical trials. However, the stated-preference design was simple (one trial feature varying in each follow-up question) due to the large number of potentially relevant trial features included. While this design provides insight into the relative importance of trial features, alternative designs that simultaneously vary a smaller number of trial features (e.g., discrete choice experiments) would further our understanding of how patients trade-off features of study designs when making participation decisions. Future studies would also benefit from involving larger samples and a broader range of countries and indications. Results generated from these studies should provide insight to sponsors for optimizing study design to aid enrolment.

## Conclusions

This study aimed to quantity the impact of study and patient characteristics on clinical study participation rates. The results suggest that those designing studies can improve participation by increasing payments to participants, reducing study duration, and decentralizing studies. These findings may not be so unexpected. However, our research quantifies these effects and how they vary with patient characteristics, raising the possibility of using such insights to simulate how changing designs will influence participation. Moreover, it was possible to identify meta-characteristics that explained why participation rates varied between indications and countries, raising the possibility that such predictions might be generalizable. Realizing these possibilities will require further participation data that allows investigation of the trade-offs patients are willing to make between key study design features**.**

## Supplementary Information

**Additional file 1.**

**Additional file 2.**

**Additional file 3.**

**Additional file 4.**

**Additional file 5.**

**Additional file 6.**

## Data Availability

The datasets generated and/or analyzed during the current study are not publicly available as they are proprietary and no consent was sought from participants to allow sharing of data with third parties beyond the current study. Requests for anonymized data can be made to the corresponding author.

## Abbreviations

BIC: Bayesian Information Criterion
OR: Odds ratio
US: United States
