# Supplementary material for: How do study design features and participant characteristics influence willingness to participate in clinical trials? Results from a choice experiment
Source: BMC Med Res Methodol. 2022 Dec 16;22:323. doi: 10.1186/s12874-022-01803-6 (PMC9756590; doi:10.1186/s12874-022-01803-6)
Supplement: Supplementary file 1 — Additional file 1. [file 12874_2022_1803_MOESM1_ESM.docx]

# Additional file 1: Targeted literature review

Targeted literature review search strings (Ovid Algorithm-Embase and Medline)

| Search Number | Search Terms | Results |
| --- | --- | --- |
| 1 | (((clinical trial or clinical trials or trial or trials or study or studies) and (recruit$ or retention or participat$ or enrol$ or willingness to participate)) or (preferences and clinical trials)).ti. | 30,898 |
| 2 | exp discrete choice experiment/ or (discrete choice or choice experiment or benefit risk analys$ or multicriteria decision analysis or stated preference$ or conjoint analysis or best-worst scaling or analytical hierarchy process or Macbeth or swing weight or thresholding or revealed preference or time trade off or standard gamble or contingent valuation or mcda or dce or bws or vignette).ti,ab. | 57,245 |
| 3 | 1 and 2 | 76 |
| 4 | exp clinical trial/ or clinical trial.pt. | 2,427,820 |
| 5 | 3 not 4 | 53 |
| 6 | (nurs$ or parent$ or doctor$ or physician$ or pharmacist$ or "barriers to treatment" or valid$ or reliab$ or challenge stud$ or challenge trial$ or challenge protocol$ or challenge test stud$ or in vitro).ti. | 2,028,809 |
| 7 | 5 not 6 | 44 |
| 8 | 7 not (animals/ not humans/) | 43 |
| 9 | 8 not ((exp animal/ or nonhuman/) not exp human/) | 42 |
| 10 | Remove duplicates from 9 | 28* |

* Due to the targeted nature of the review, the 28 eligible papers were reviewed and 20 papers were extracted, prioritizing a diversity of indications and methods.


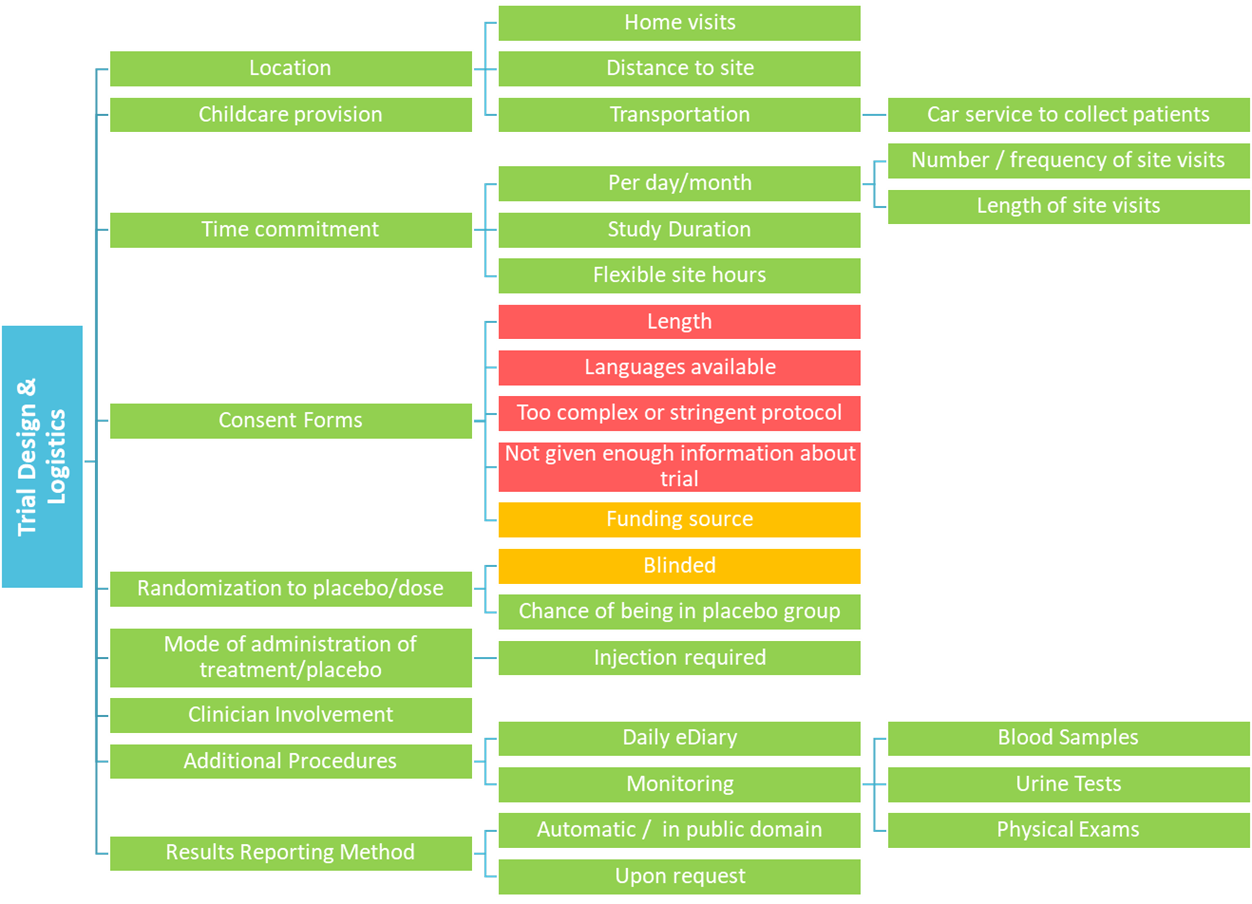


**Figure S1. Trial design- and logistics-related attributes identified from the targeted literature review.** Features identified from the targeted literature review are organized into four groups: green, features within the control of trial design team and relevant to our study; yellow, features likely fixed in any study design and thus beyond the scope of this study; red, features beyond the influence of trial design teams and thus beyond the scope of this study.


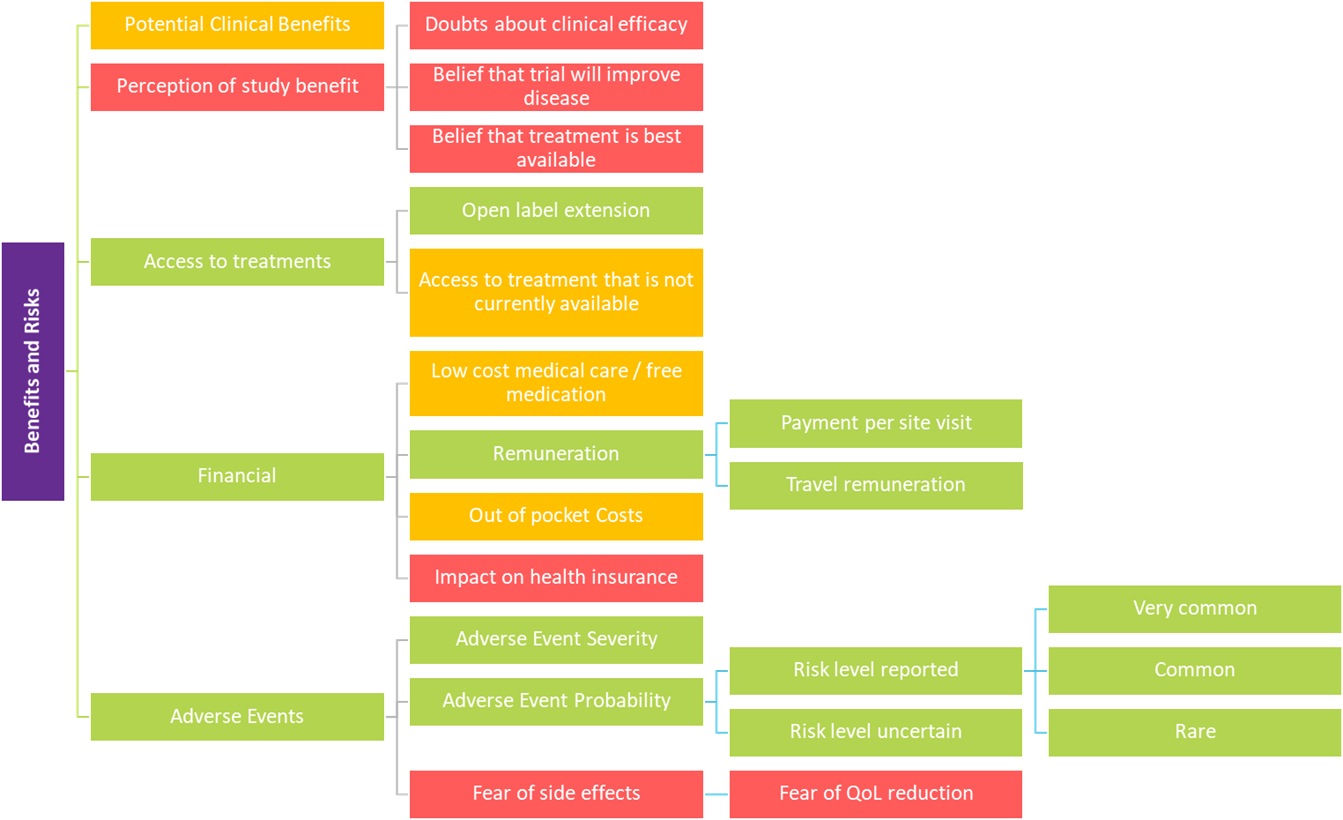


**Figure S2. Benefits and risk attributes identified from the targeted literature review.** Features identified from the targeted literature review are organized into four groups: green, features within the control of trial design team and relevant to our study; yellow, features likely fixed in any study design and thus beyond the scope of this study; red, features beyond the influence of trial design teams and thus beyond the scope of this study.


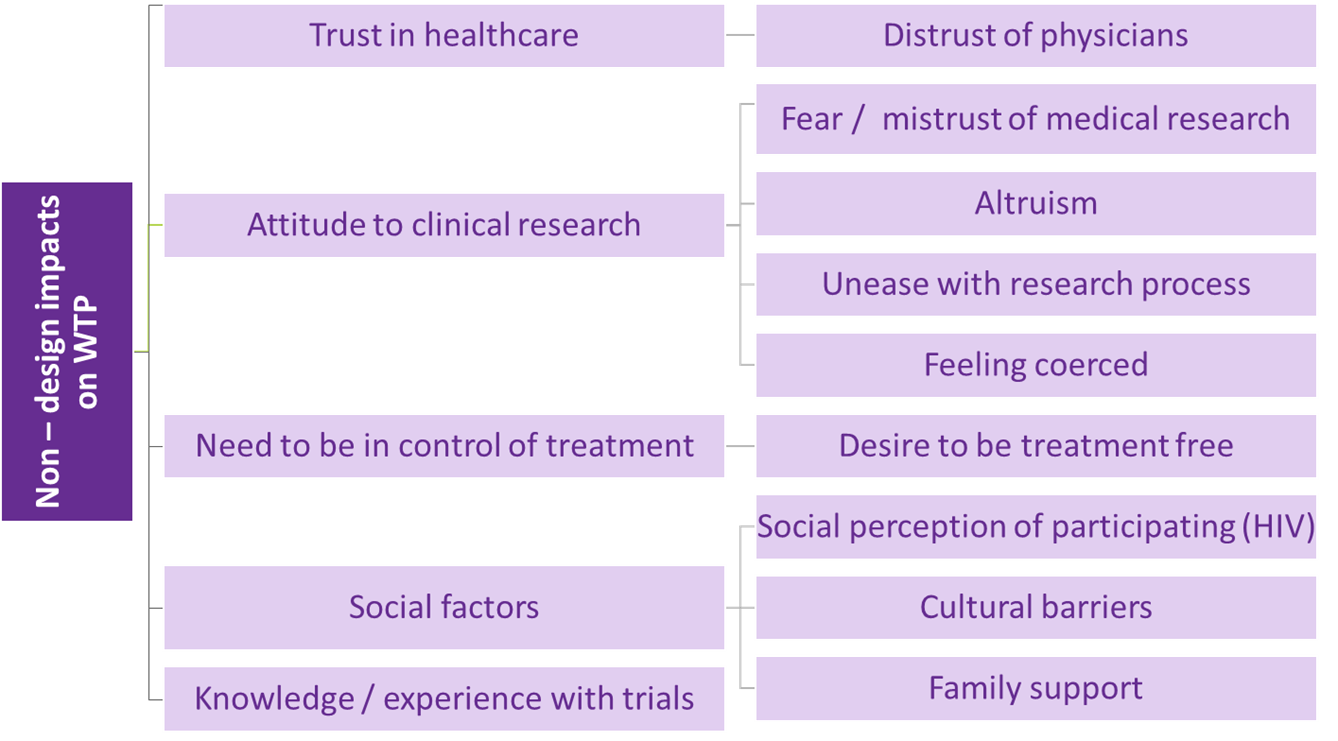


**Figure S3. Attitude, knowledge, and social context features impacting willingness to participate (WTP) identified from the targeted literature review.** These features informed the selection of participant characteristics to collect data on in our study (e.g., altruism and trust in researchers)
