# Supplementary material for: How do study design features and participant characteristics influence willingness to participate in clinical trials? Results from a choice experiment
Source: BMC Med Res Methodol. 2022 Dec 16;22:323. doi: 10.1186/s12874-022-01803-6 (PMC9756590; doi:10.1186/s12874-022-01803-6)
Supplement: Supplementary file 2 — Additional file 2. [file 12874_2022_1803_MOESM2_ESM.docx]

# Additional file 2

**Table S1. Trial design feature definitions provided to participants during the survey**

| **Category** | **Clinical Study Feature** | **Definition** |
| --- | --- | --- |
| **Payment and support** | **Payment you will receive** | Some studies compensate participants for their time and contribution. The amounts shown are the total amount you will be paid for completing all parts of the study. |
|  | **Transport** | Transport costs of being in a study will be covered by the study sponsor. How this is done varies. In some studies transport is provided, for example a prepaid taxi is arranged for you. In other studies, you will have to pay for your own transport but can claim back the cost afterwards. Other studies provide you with a prepaid debit card that can use to pay for transport. |
|  | **Study Hours** | Some studies require your participation during office hours (i.e. 9am to 5pm Monday to Friday). Other studies provide the option to participate out of office hours (i.e. also in the evening or on the weekend). |
|  | **Childcare** | Some studies will provide free childcare if you are at a study visit. Other studies do not provide childcare and would need to make your own childcare arrangements. |
|  | **Concierge Service Provided** | Some studies provide a concierge service to support your participation. This would include:  Sending you reminders when you to take your medication or participate in the study.  A phone number you can call if you have any questions or concerns during the study, such as if you need help with study technology.  In other studies, you would have to contact the study site or an IT helpline directly for the above support. |
| **Administration / Procedures** | **Does treatment or study require an injection or infusion?** | Some studies involve treatments or data collection that require injections, blood draws and/or infusions. You may experience some injection site pain. |
|  | **Does treatment or study require an invasive procedure?** | Some studies may require a procedure.  Minimally invasive procedures include colonoscopies, keyhole surgery etc. These will require you to attend hospital for less than one day and be sedated for one hour for the procedure. You may feel some pain at the procedure site, which can be managed with over the counter pain medicine. You can resume normal activities a few days later.  Invasive procedures involve you being sedated for 4 hours and staying at the hospital for 1 week. You will not be able to resume normal activities for 1 month following the procedure. |
| **Treatment-related** | **Number of participants who will have a serious side effect** | Different treatments have different risks of serious side effects. Serious side effects will require hospitalization and in rare cases may be fatal. |
|  | **Number of participants who will receive a placebo** | A placebo is a product that does not have the active ingredient to treat any condition. Some studies give a placebo to a proportion of participants. If this is the case, you will not know if you are given the placebo or the study treatment. |
|  | **Is it possible to continue on treatment after trial?** | On completion of the initial study you may be invited to continue to take the study drug for an extended period. If not, you will return to your current treatment at the end of the study. |
|  | **Are you required to stop using your current medications?** | Some studies require you to stop taking your existing medications. Other studies allow you to take the study treatment in addition to your existing medications. |
| **Study location and time commitment** | **Study duration** | Clinical studies are conducted for different periods of time. Throughout the study duration you would be required to attend study appointments. |
|  | **Time commitment per month at home** | The amount of time per month you spend participating in the study at your home. This includes time for home visits, virtual tele visits and time for completing questionnaires. |
|  | **Time commitment per month away from home (i.e. time at study site and travel to site)** | The amount of time per month you spend at a study site or getting to the study site. |
| **Data collection and feedback** | **Does the study involve wearing a device?** | Some studies require you to wear a small device. These are portable and collect data using sensors, such as your activity levels or the amount your sleep. |
|  | **How will you provide self-reported data?** | All studies will require you to complete questionnaires. This can either be done on a paper version of the questionnaire or completed using an electronic device or app. |
|  | **Will you be told the results of the study?** | Some studies provide participants with the results of the study. This can be either the average improvement in health across all participants or your own improvement in health. |
