# Supplementary figures and images for: How do study design features and participant characteristics influence willingness to participate in clinical trials? Results from a choice experiment
Source: BMC Med Res Methodol. 2022 Dec 16;22:323. doi: 10.1186/s12874-022-01803-6 (PMC9756590; doi:10.1186/s12874-022-01803-6)

# Additional file 3

**
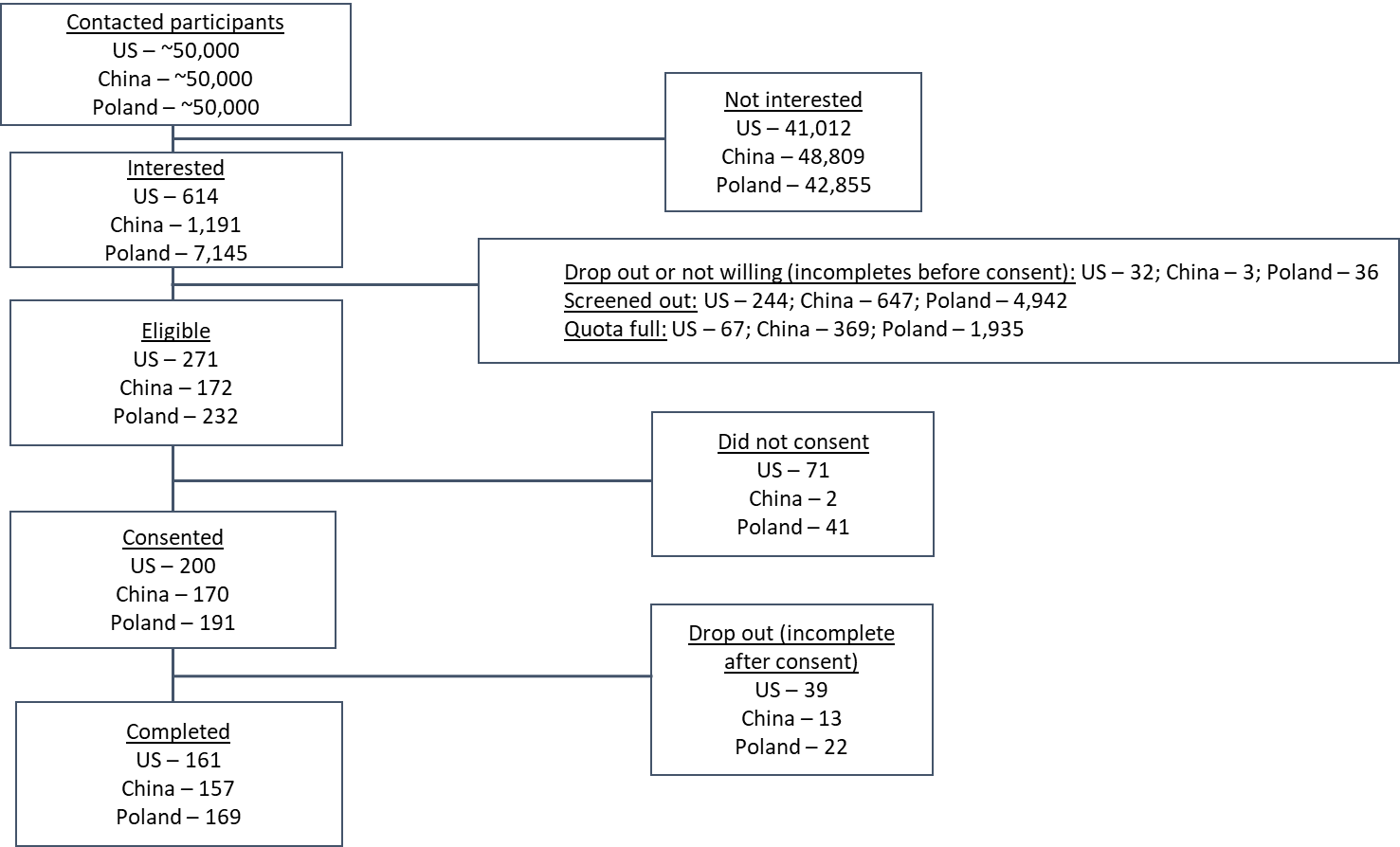
**

**Figure S4. Disposition diagram**

Supplement: Supplementary file 3 — Additional file 3. [file 12874_2022_1803_MOESM3_ESM.docx]
