# Supplementary material for: How do study design features and participant characteristics influence willingness to participate in clinical trials? Results from a choice experiment
Source: BMC Med Res Methodol. 2022 Dec 16;22:323. doi: 10.1186/s12874-022-01803-6 (PMC9756590; doi:10.1186/s12874-022-01803-6)
Supplement: Supplementary file 4 — Additional file 4. [file 12874_2022_1803_MOESM4_ESM.docx]

# Additional file 4

**Table S2. Large and moderate correlations between personal characteristics**

| **Characteristic 1** | **Characteristic 2** | **Absolute coefficient** | **Classification ^a^** |
| --- | --- | --- | --- |
| Country | Age Group | 0.63 | Large |
| Employment Status | Age Group | 0.61 | Large |
| Employment Status | Country | 0.54 | Large |
| Self-perceived future quality of life | Health compared to one year ago | 0.46 | Moderate |
| Health compared to others | Self-rated general health | 0.45 | Moderate |
| Living status and caring responsibilities | Age Group | 0.44 | Moderate |
| Satisfaction with current disease management | Age Group | 0.41 | Moderate |
| Satisfaction with current disease management | Employment Status | 0.41 | Moderate |
| Satisfaction with current disease management | Country | 0.39 | Moderate |
| Living status and caring responsibilities | Country | 0.37 | Moderate |
| Side effects from treatments | Previous treatments taken | 0.37 | Moderate |
| Living status and caring responsibilities | Employment Status | 0.36 | Moderate |
| Health compared to others | Likelihood that you will live for the next years or longer | 0.36 | Moderate |
| Health compared to others | Self-perceived future quality of life | 0.36 | Moderate |
| Self-rated general health | Country | 0.35 | Moderate |
| Satisfaction with current disease management | Self-rated general health | 0.35 | Moderate |
| Self-perceived future quality of life | Likelihood that you will live for the next years or longer | 0.35 | Moderate |
| Altruism | Trust in medical researchers | 0.34 | Moderate |
| Self-perceived future quality of life | Age Group | 0.31 | Moderate |
| Medications affordability | Self-rated general health | 0.31 | Moderate |
|  | | | |

^a^ Based on Cohen (1988) classification: very small, 0.0–0.1; small, 0.1–0.3; moderate, 0.3–0.5; large, 0.5–0.7; very large, 0.7–0.9; almost perfect, 0.9–1.0
