# Supplementary material for: How do study design features and participant characteristics influence willingness to participate in clinical trials? Results from a choice experiment
Source: BMC Med Res Methodol. 2022 Dec 16;22:323. doi: 10.1186/s12874-022-01803-6 (PMC9756590; doi:10.1186/s12874-022-01803-6)
Supplement: Supplementary file 5 — Additional file 5. [file 12874_2022_1803_MOESM5_ESM.docx]

# Additional file 5

**Table S3. Additional participant demographics and baseline characteristics**

| **Characteristic** | **Overall (N = 487)** |
| --- | --- |
| **Living status and caring responsibilities, n (%)** |  |
| Living Alone | 58 (12) |
| Living with others (spouse, partner, relatives or friend) | 209 (43) |
| Looking after any dependent family members (for example children, elderly or disabled relatives) | 214 (44) |
| Prefer not to say | 6 (1) |
| **Remission status, n (%)** |  |
| In remission | 135 (74) |
| Not in remission | 48 (26) |
| **Time since diagnosis (years)** |  |
| Mean (SD) | 8 (9) |
| Min–Max | 0 – 61 |
| **Affordability of current medication, n (%)** |  |
| I can easily afford my medications | 168 (34) |
| I can afford my medications | 272 (56) |
| I struggle to pay for my medications | 47 (10) |
| **I am as healthy as anybody I know, n (%)** |  |
| Definitely true | 41 (8) |
| Mostly true | 143 (29) |
| Don't know | 86 (18) |
| Mostly false | 153 (31) |
| Definitely false | 64 (13) |
| **QoL vs. last year, n (%)** |  |
| Much better now than one year ago | 46 (9) |
| Somewhat better now than one year ago | 113 (23) |
| About the same | 227 (47) |
| Somewhat worse now than one year ago | 85 (17) |
| Much worse now than one year ago | 16 (3) |
| **COVID-19 experience/concern, n (%)** |  |
| Had a positive COVID-19 test with mild to moderate symptoms | 23 (5) |
| Had a positive COVID-19 test with severe symptoms that required hospitalization | 25 (5) |
| Had a positive antibody test | 427 (88) |
| I have not had COVID-19/ or had mild symptoms but not been tested | 12 (2) |
| Not sure / Prefer not to answer | 0 (0) |
| **COVID-19 vaccination, n (%)** |  |
| Yes | 223 (46) |
| No | 264 (54) |

Abbreviations: QoL, quality of life.
Education levels: Less than elementary/primary school includes *no formal qualifications*; Elementary / primary school = Less than high school/technical school or equivalent; High school / technical school or equivalent includes post-secondary
