# Supplementary material for: How do study design features and participant characteristics influence willingness to participate in clinical trials? Results from a choice experiment
Source: BMC Med Res Methodol. 2022 Dec 16;22:323. doi: 10.1186/s12874-022-01803-6 (PMC9756590; doi:10.1186/s12874-022-01803-6)
Supplement: Supplementary file 6 — Additional file 6. [file 12874_2022_1803_MOESM6_ESM.docx]

# Additional file 6

Table S4. Preferences for attributes (latent class logit results)

| **Characteristics** | **Levels** | **Change** |  | **Overall (Shift: 25%)** | |  | **Class 1 (Share: 36.1%; Shift: 55%)** | |  | **Class 2 (Share: 63.9%; Shift: 8%)** | |
| --- | --- | --- | --- | --- | --- | --- | --- | --- | --- | --- | --- |
|  |  |  |  | **MLE (SE)** | **OR** |  | **MLE (SE)** | **OR** |  | **MLE (SE)** | **OR** |
| Constant | - | - |  | - | - |  | **-1.075 (0.327)^***^** | 0.34 |  | -**3.803 (0.949)^***^** | 0.02 |
| Survey version | Version 1 | - |  | Reference | - |  | - | - |  | - | - |
|  | Version 2 | - |  | - | - |  | -0.036 (0.198) | 0.96 |  | **0.484 (0.283)^*^** | 1.62 |
| Feature modification | Improvement | - |  | Reference | - |  | - | - |  | - | - |
|  | Deterioration | - |  | - | - |  | **0.496 (0.225)^**^** | 1.64 |  | -0.159 (0.313) | 0.85 |
| Trial features | Transport provision | 22/191 (11.5%) |  | Reference | - |  | - | - |  | - | - |
|  | Serious side effect | 85/185 (45.9%) |  | 2.468 (0.543)^***^ | 11.80 |  | **2.202 (0.487)^***^** | 9.04 |  | **2.631 (0.927)^***^** | 13.89 |
|  | Payment | 74/189 (39.2%) |  | 2.033 (0.541)^***^ | 7.64 |  | **1.998 (0.480)^***^** | 7.37 |  | **2.055 (0.944)^**^** | 7.81 |
|  | Stop medication | 28/89 (31.5%) |  | 1.710 (0.580)^***^ | 5.53 |  | 0.507 (0.511) | 1.66 |  | **2.447 (0.928)^***^** | 11.56 |
|  | Invasive procedure | 66/191 (34.6%) |  | 1.410 (0.590)^**^ | 4.10 |  | **1.964 (0.453)^***^** | 7.13 |  | 1.070 (1.017) | 2.92 |
|  | Study duration | 46/165 (27.9%) |  | 1.289 (0.561)^**^ | 3.63 |  | **1.079 (0.422)^**^** | 2.94 |  | 1.419 (0.948) | 4.13 |
|  | Study hours | 25/99 (25.3%) |  | 1.035 (0.633) | 2.82 |  | **1.081 (0.504)^**^** | 2.95 |  | 1.007 (1.076) | 2.74 |
|  | Injection | 16/93 (17.2%) |  | 0.985 (0.599)^*^ | 2.68 |  | -0.035 (0.621) | 0.97 |  | 1.611 (0.990) | 5.01 |
|  | Time commitment away from home | 61/202 (30.2%) |  | 0.873 (0.779) | 2.39 |  | **2.471 (0.596)^***^** | 11.83 |  | -0.106 (1.297) | 0.90 |
|  | Results sharing | 38/199 (19.1%) |  | 0.487 (0.655) | 1.63 |  | **0.830 (0.406)^**^** | 2.29 |  | 0.276 (1.103) | 1.32 |
|  | Placebo | 33/189 (17.5%) |  | 0.453 (0.638) | 1.57 |  | 0.410 (0.415) | 1.51 |  | 0.480 (1.085) | 1.62 |
|  | Wear device | 13/96 (13.5%) |  | 0.405 (0.638) | 1.50 |  | -0.144 (0.544) | 0.87 |  | 0.742 (1.070) | 2.10 |
|  | Concierge service | 15/101 (14.9%) |  | 0.272 (0.753) | 1.31 |  | 0.463 (0.485) | 1.59 |  | 0.155 (1.247) | 1.17 |
|  | Continue treatment | 15/88 (17%) |  | 0.175 (0.922) | 1.19 |  | 0.645 (0.493) | 1.91 |  | -0.113 (1.527) | 0.89 |
|  | Time commitment at home | 46/179 (25.7%) |  | 0.132 (1.075) | 1.14 |  | **1.223 (0.410)^***^** | 3.40 |  | -0.537 (1.782) | 0.58 |
|  | Childcare | 11/82 (13.4%) |  | -0.136 (0.945) | 0.87 |  | 0.181 (0.536) | 1.20 |  | -0.330 (1.579) | 0.72 |
|  | Self-report data | 13/97 (13.4%) |  | -1.030 (2.934) | 0.36 |  | 0.181 (0.511) | 1.20 |  | -1.773 (4.825) | 0.17 |

Analysis information: Respondents= 487; Observations= 2435; Parameters= 39; LL= -1157.2; BIC= 2618.6; APR= 29.13%
Abbreviations: LL= Log-likelihood; BIC=Bayesian information criteria; APR=Adjusted McFadden Pseudo-R2; MLE= Maximum likelihood estimate; SE= Standard error; OR= Odds ratio. *p<0.1; **p<0.05; ***p<0.01
